# Constant Multi-Tasking With Time Constraint to Preserve Across-Network Dynamics Throughout Awake Surgery for Low-Grade Glioma: A Necessary Step to Enable Patients Resuming an Active Life

**DOI:** 10.3389/fonc.2022.924762

**Published:** 2022-05-26

**Authors:** Hugues Duffau, Sam Ng, Anne-Laure Lemaitre, Sylvie Moritz-Gasser, Guillaume Herbet

**Affiliations:** ^1^ Department of Neurosurgery, Gui de Chauliac Hospital, Montpellier University Medical Center, Montpellier, France; ^2^ Team “Plasticity of Central Nervous System, Stem Cells and Glial Tumors”, U1191 Laboratory, Institute of Functional Genomics, National Institute for Health and Medical Research (INSERM), University of Montpellier, Montpellier, France; ^3^ Department of Speech-Language Pathology, University of Montpellier, Montpellier, France

**Keywords:** awake brain surgery, brain connectome, cognitive monitoring, electrostimulation mapping, low-grade glioma, multitasking, neural networks, neuroplasticity

## Abstract

Awake surgery for brain gliomas improves resection while minimizing morbidity. Although intraoperative mapping was originally used to preserve motor and language functions, the considerable increase of life expectancy, especially in low-grade glioma, resulted in the need to enhance patients’ long-term quality of life. If the main goal of awake surgery is to resume normal familial and socio-professional activities, preventing hemiparesis and aphasia is not sufficient: cognitive and emotional functions must be considered. To monitor higher-order functions, e.g., executive control, semantics or mentalizing, further tasks were implemented into the operating theater. Beyond this more accurate investigation of function-specific neural networks, a better exploration of the inter-system communication is required. Advances in brain connectomics led to a meta-network perspective of neural processing, which emphasizes the pivotal role of the dynamic interplay between functional circuits to allow complex and flexible, goal-directed behaviors. Constant multi-tasking with time constraint in awake patients may be proposed during intraoperative mapping, since it provides a mirror of the (dys)synchronization within and across neural networks and it improves the sensitivity of behavioral monitoring by increasing cognitive demand throughout the resection. Electrical mapping may hamper the patient to perform several tasks simultaneously whereas he/she is still capable to achieve each task in isolation. Unveiling the meta-network organization during awake mapping by using a more ecological multi-demand testing, more representative of the real-life conditions, constitutes a reliable way to tailor the surgical onco-functional balance based upon the expectations of each patient, enabling him/her to resume an active life with long-lasting projects.

## Introduction

Early and maximal safe surgical resection is currently the first treatment in low-grade glioma (LGG) patients, since it drastically extends the overall survival [now above 16 years (1, 2)] while minimizing the risk of lasting neurological deficit [almost nil in recent series (3, 4)]. To this end, intraoperative direct electrostimulation (DES) mapping is considered as the gold standard in glioma surgery (5), especially when achieved in awake patients with real-time cognitive mapping and monitoring throughout the resection in order to identify and spare the neural connectivity (6). However, although intrasurgical mapping was so far mostly used to preserve motor and language functions, the significant increase of survival in LGG patients resulted in the need to further optimize the quality of life (QoL) in the long run (7). Therefore, if the main goal of awake surgery is to allow patients to resume normal familial and socio-professional activities, preventing hemiparesis and aphasia is clearly not sufficient: cognitive and emotional functions should also be considered (8). Further tasks have recently been added into the operating theater, in order to monitor higher-order functions such as executive control, semantics, or mentalizing (8–10). Nonetheless, beyond this more accurate investigation of function-specific neural networks, a better exploration of the inter-system communication across multiple integrated networks is also required. Indeed, advances in the science of brain connectomics led to a meta-network perspective of neural processing, which emphasizes the pivotal role of the dynamic interplay between functional circuits to allow flexible adaptation to the environment (11).

With that in mind, the purpose is here to consider the use of constant multi-tasking with time constraint in awake patients during intraoperative cognitive mapping, since it provides a mirror image of the (dys)synchronization within and across neural networks and it improves the sensitivity of the behavioral monitoring in real-time by increasing the cognitive demand. The ultimate aim of such a paradigmatic shift in brain mapping, which consists in incorporating more ecological tasks throughout the resection, adapted to the needs of each patient, is to preserve the complex behavior necessary to enjoy a normal life.

## The Need to Improve Long-Term Functional Outcomes in LGG Patients: Towards A Longitudinal Evaluation of Cognition

With the increase of life expectancy in LGG patients, more attention should be paid to their QoL over years, including their ability to make long-term familial and professional projects (12, 13). For this purpose, movement and conation (i.e., the willingness which leads to action) (14), cognition (such as language, executive functions, attention, memory or semantic processing) (15, 16), metacognition (knowing of knowing) (17), emotion, personality and behavior (18) should be carefully assessed in routine before and after each treatment in LGG patients. Indeed, because they are usually young and lead an active life at diagnosis, with no deficit at so-called “standard neurological examination”, it has usually been considered that these patients did not experience functional disturbances (except possible seizures) (19). Yet, a recent series with 157 LGG patients who benefited from an extensive preoperative neuropsychological assessment evidenced that 55.4% of them already had cognitive impairments, especially regarding language, verbal episodic memory, psychomotor speed and attention, as well as executive functions (phonological and categorical fluency) (4). Of note, neurocognitive declines have even been observed in patients with an incidental discovered LGG (20). Interestingly, specific-domain deficits have been correlated to the glioma infiltration of the neural pathways underlying the corresponding functions, e.g., verbal semantic deterioration linked to the invasion of the left inferior fronto-occipital fasciculus (21) or visuo-spatial impairments linked to the invasion of the right superior longitudinal fasciculus (22).

Moreover, for many decades, only motor and language outcomes have been studied following glioma resection. In fact, even though intraoperative electrostimulation mapping has been shown to significantly decrease the rate of persistent hemiparesis and/or aphasia (5), higher-order functions have received less attention. However, recent studies have evidenced a frequency of cognitive declines higher than previously thought after glioma surgery, in one or several domains such as language, attention processing, executive functions and verbal memory (16, 23, 24) - including after tumor resection within so-called “non-eloquent” brain areas (25). In addition, postoperative mentalistic deficits and behavioral or personality changes have also been observed (18, 26, 27). Remarkably, these impairments have been correlated with the neural structures damaged by either surgical gesture or residual tumor infiltrations; for example, picture naming worsening and damage to the left ILF (28), impairment of mental flexibility and injury to the SLF (10), decline of mentalizing abilities and damage of the right arcuate fasciculus and/or cingulum (29), or heightened schizotypal traits and lesion of the left uncinate fasciculus (30). Above all, not only postsurgical scores but also reaction times have a predictive value regarding the return to a normal life: indeed,lexical access speed was significantly linked to the capacity to resume professional activities (31). Thus, to improve postoperative outcomes, beyond new programs of neurocognitive rehabilitation aiming at facilitating the recovery of higher-order functions such as attention, memory or mental fatigue (32–34), a paradigmatic shift in the intraoperative functional mapping “à la carte” has recently been proposed (9).

## The Recent Refinement of Intraoperative Cognitive Monitoring Using Specific-Domain Tasks

Based upon the results of postsurgical objective neuropsychological assessments, with the goal of minimizing the risk to generate long-lasting cognitive and emotional disturbances, further tasks have been introduced during DES mapping in awake patients in order to refine the functional monitoring of higher-order functions throughout the resection (8). A comprehensive review of the current literature has recently been published by Mandonnet and Herbet (35), who showed that, besides the typical motor as well as spoken and written language testing, additional functions can be mapped into the operating room, such as visuo-spatial functions (36), multimodal (verbal and non-verbal) semantics (14, 37), executive functions (e.g., working memory, set shifting, inhibition) (10), social cognition (38) or even self-evaluation (39), and so on.

The selection of tasks can be made based upon several criteria in order to perform an optimal cortical and subcortical DES mapping, namely, according to the results of the preoperative neuropsychological assessment (4), the location of the tumor, including the relationships between the glioma and surrounding functional pathways (40), the economic and socio-cultural environment (41), and most of all the characteristics of the patient (age, education) as well as his/her wishes – i.e., his/her definition of QoL, taken into account his/her job, hobbies, lifestyle, daily activities, and so forth (9). Nonetheless, despite such an improvement of the real-time intrasurgical mapping, each task is dedicated to monitor exclusively or mainly one functional system subserving one given cognitive domain. Yet, daily complex behaviors are performed in a specific environmental, semantic and social context and thus requires the flexible integration of multiple cognitive representations (11). As a consequence, with the aim of tending towards more naturalistic tasks representative of real-life conditions, multi-demand testing may be considered for intrasurgical mapping.

## The New Concept of Constant Multi-Tasking with Time Constraint Throughout the Surgical Resection

In fundamental neurosciences, new network models of cognition not only broke with the traditional localizationist view (one cerebral area sustaining one specific function), but went beyond a simple network distribution of the brain (one cerebral circuit sustaining one specific function) by emphasizing the pivotal role of inter-system communication in the service of complex behaviors - which are adapted to the ever-changing surrounding world (11). In agreement with this meta-networking organization, complex cognitive processes (e.g., multitasking abilities) may be the result of a recruitment and coordination (combination or even competition) of widespread neural resources not only involving domain-specific networks (e.g., language or motor networks), but also a multiple-demand system which is activated during performances of a wide range of cognitive-demanding activities in order to maintain fluid intelligence (42, 43). This multiple-demand network, composed of several brain structures, notably the lateral frontal cortex, the anterior insula, the frontal operculum, the supplementary motor area, the anterior cingulate cortex and the cortex within the intraparietal sulcus (44), is recruited for more demanding tasks, including multi-tasking (45) and time pressure (46).

Therefore, to benefit from a mirror of this dynamic interaction across neural circuits, intraoperative multi-tasking has recently been introduced in awake patients throughout the tumor resection (39, 47). Of note, it has previously been proposed to alternate several tasks in order to preserve language and/or other-than-language cognitive functions, but in a rotating and sequential manner (i.e., object naming task followed by word reading, then followed by word repetition, and so forth) (48, 49). Nonetheless, beyond the fact that this is actually not a combined multi-tasking, since tasks are applied in serial order (thus with limited cognitive demand), the risk of a fixed sequence is to administrate a test not specifically appropriate to the region operated on in real-time – i.e., a test not adapted to monitor the specific subnetwork(s) surrounding the surgical resection performed at that time, thus decreasing the reliability of the functional mapping. In fact, Skrap et al. (48) wrote that “For example, in each test for a patient who underwent surgery in the left temporal lobe, 10–15 items were presented (for instance, 15 pictures for naming, 10 words for reading, 10 pseudo-words for repetition) for about 30 seconds for each task, in a rotating manner.” Yet, in this example, although the use of a reading task seems accurate to deal with the posterior part of the tumor [since reading deficits could be generated into the contact of the posterior inferior longitudinal fasciculus and the arcuate fasciculus nearby the visual word form area (50, 51)], this task is not appropriate to monitor and spare the fibers of the inferior fronto-occipital fasciculus at the upper and deeper part of the temporal tumor [a white matter tract devoted to multimodal semantic processing (14, 52)]. Finally, Colle et al. have more recently proposed to administrate multiple tasks in awake patients, but only for mapping sub-functions of language (53).

Here, the goal is to propose a more ecological testing philosophy, more representative of the needs required in the real-life for each patient according to her/his expectations, based upon the following criteria:

- A constant multi-tasking combining several tests performed simultaneously, during the transitory presentation of a problem to solve on a computer screen while allowing to stimulate a specific brain structure in this time window, e.g., movement combined with semantic association task (such as Pyramid and palm-tree test, PPTT) (54) while naming the pictures of the PPTT; or a mentalizing task (Read the Mind in the Eyes task, RME) (55) with an index reflecting the level of confidence of the patient in his/her own response (ranging from 1 to 6, 6 being the most confident response) (39) combined with movement; and so forth;
- With a time constraint, that is, items should change every 4-5 seconds, without any rest throughout the resection;
- Switching from a combination of several tasks to another combination of other tests depending on the various cortical and subcortical structures successively encountered throughout resection, i.e., according to the proximity of the different neural networks surrounding the surgical cavity.

**Figure 1:**
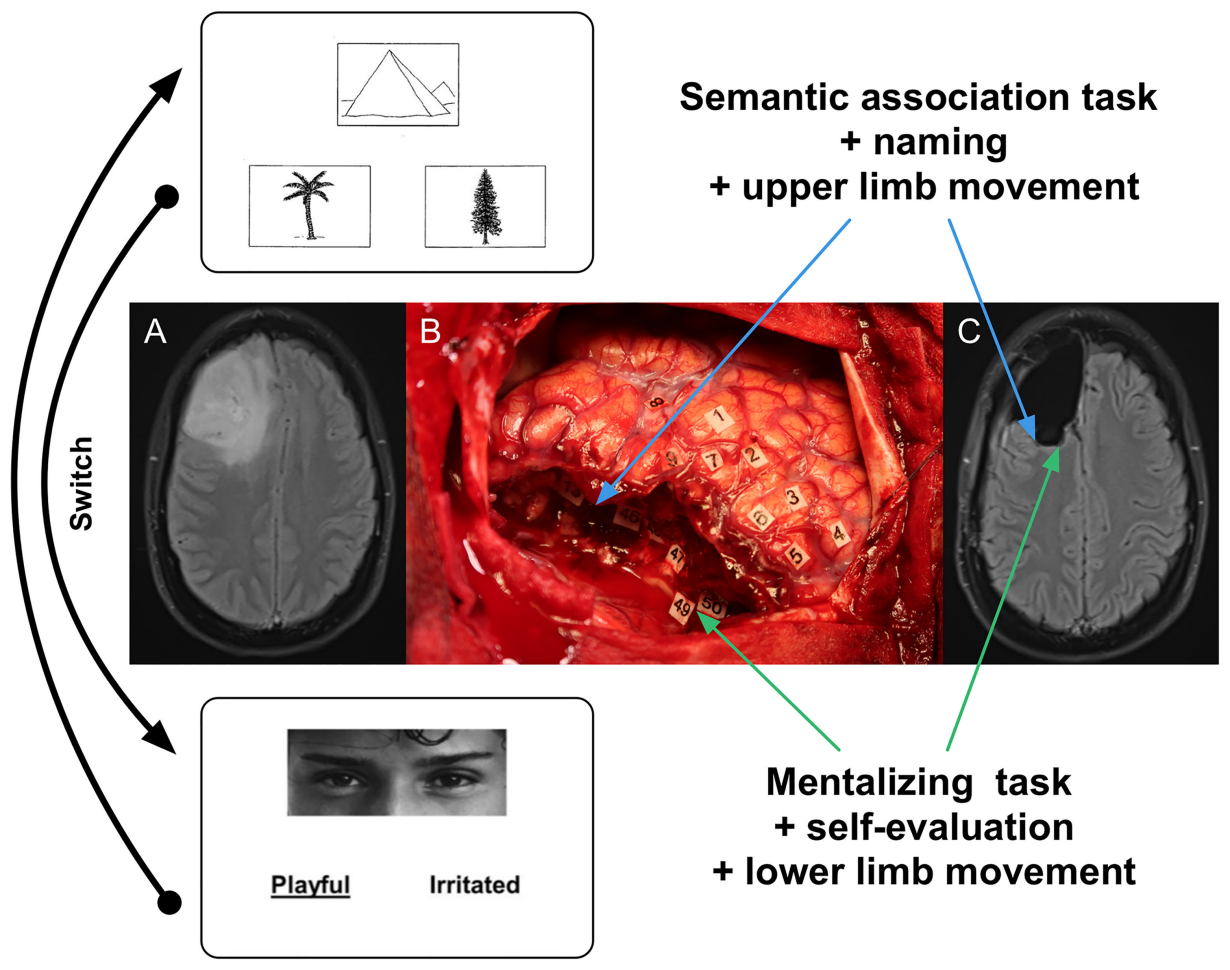
Illustration of intraoperative constant multi-tasking adapted to the surrounding neural networks encountered throughout the resection. **(A)** Axial FLAIR-weighted MRI showing a right frontal LGG discovered in a 55-year right-handed man who experienced seizures. The preoperative neurological and cognitive assessments were normal. **(B)** Intraoperative view after surgical resection achieved up to functional boundaries in awake patient (the anterior part of the brain is on the left and its posterior part is on the right). Number tags show zones of positive DES mapping (2 mA) as follows: - tags 1, 2 and 8: ventral premotor cortex (lateral part of the precentral gyrus) generating anarthria during DES; - tag 3: primary motor cortex of the face, inducing involuntary facial movements during DES; - tag 4 and 5: primary motor cortex of the left upper limb, eliciting involuntary movements during DES; - tags 6 and 7: negative motor sites (anterior part of the precentral gyrus) generating arrest of both speech and movement of the left upper limb during DES; - tag 9: area involved in semantic processing, evoking PPTT (semantic association task) disturbances during DES. Subcortically, the resection was also performed according to critical networks detected in a patient achieving constant multi-tasking. First, the patient was asked to perform motor task combined with PPTT when removing the postero-lateral part of the tumor close to the frontal terminations of the inferior fronto-occipital fasciculus (tag 13, inducing semantic deficit during DES) as well the junction between the fronto-striatal tract (tags 46 and 48, generating arrest of movement during DES) and the pyramidal pathway (tag 47, eliciting involuntary movement of the left upper limb during DES) (blue arrows). Then, the patient was asked to switch to a mentalizing task (RME) combined with a self-evaluation test and a movement of the lower limb when removing the postero-mesial and deep part of the lesion close to the cingulate bundle: DES of the fronto-striatal tract (medial portion) induced arrest of movement of the lower limb (tag 50) while DES of the cingulate fibers generated mentalistic deficits in a patient still able to move the lower limb (tag 49) (green arrows). **(C)** Axial FLAIR-weighted MRI performed 3 months after surgery, revealing a complete resection, in a patient able to resume an active familial and socio-professional life with neither neurological nor neurocognitive deficit. Blue and green arrows show the correlations between functional subcortical structures which induced transitory behavioral disturbances during DES and their anatomical positions on the postoperative MRI.

As an illustration, considering the resection of a right frontal tumor, the motor task can be combined with PPTT when removing the postero-lateral part of the lesion close to the frontal terminations of the inferior fronto-occipital fasciculus and the junction between the fronto-striatal tract and the pyramidal pathway; then the patient is asked to switch to a mentalizing task combined with a self-evaluation test and a movement of the lower limb when removing the postero-mesial and deep part of the lesion close to the cingulate bundle (**Figure 1**).

## Practical Implications of This Novel Intraoperative Mapping Paradigm

The first advantage of this multimodal mapping paradigm based upon the constant performance of a combination of several tasks is to enable a real-time cognitive monitoring, with a reduced risk not to test the appropriate function(s) according to the network(s) encountered throughout the resection. Moreover, the increased cognitive demand requested by multi-tasking with time pressure results in a more sensitive neuropsychological assessment into the operating theater.

**Figure 2:**
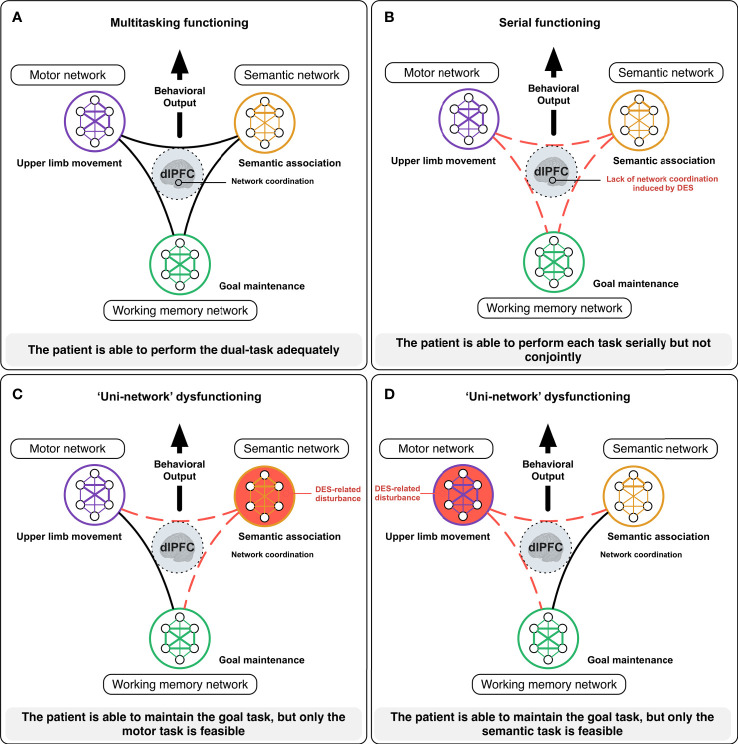
Illustration of how DES mapping may inform on the meta-networking functioning of the brain [from (11)]. During complex cognitive activities, such as multi-tasking, the brain needs to coordinate its networks to reach the task goal. In awake surgery, the patient is here asked to achieve a dual-task consisting in performing a complex movement of the upper limb in concert with a semantic association task (PPTT). In normal circumstances **(A)**, the neural activity from the semantic (semantic processing), the motor (motor initiation, control, and execution) and the working memory (goal maintenance) networks needs to be integrated to perform the task efficiently thanks to highly integrative hubs such as the dorsolateral prefrontal cortex (dlPFC). When the dlPFC is impaired by DES **(B)**, the brain has difficulties to coordinate its networks and the patient is only able to perform the tasks serially but not conjointly. When the semantic network is impaired by DES **(C)**, the patient is able to maintain the task goal, but only the motor task is performed. By contrast, when the motor network is disturbed **(D)**, the patient is still able to maintain the task goal, but only the semantic task is performed.

In practice, during resection, if the patient is not able to achieve one of the tests anymore (even if the other tests are still successfully performed) and/or if he/she needs more time to perform effective multi-tasking, the information is immediately transmitted to the neurosurgeon who can adapt the surgical strategy accordingly – without waiting for more significant functional deteriorations. Beyond cognitive monitoring, this is also true concerning DES functional mapping *per se*. Recently, a 3-level model of neural disruption using cortical-subcortical electrostimulation has been proposed (56). Besides the DES of an input/output unimodal network generating “positive” responses (as involuntary movement) (first level) and DES of a distributed specialized circuit eliciting a within-system impairment leading to specific “negative” disturbances (such as an isolated language deficit without any other impairment) (second level), DES might also induce an inter-system disruption resulting in the transient incapability to achieve multi-tasking – whereas each function can be achieved separately (third level) (56). As mentioned, in the framework of a meta-networking processing of neural functions, multimodal integration must transiently be created to succeed in complex multi-determined behaviors (11). Typically, during multi-tasking, the brain has to coordinate several networks to reach the task goal. During surgery, if the patient is regularly asked to perform a movement of the upper limb simultaneously with a picture-naming task, an interplay between the neural circuits involved in language (semantics, phonology, articulation), motor (movement control and execution), and executive functions (goal maintenance) is necessary to achieve multi-tasking efficiently thanks to transmodal cortical hubs such as the dorsolateral prefrontal cortex (56). If the language network is disrupted by DES, the patient may be able to maintain the task goal, but only movement is achieved; conversely, when the motor circuit is impaired, only the picture-naming is performed. The third level of DES disruption consists of transitory disruptions in the spatiotemporal dynamic inter-communication across function-specific circuits (e.g., by stimulating the dorsolateral prefrontal area), explaining changes in more complex multi-demand cognition: the brain cannot coordinate its networks and the patient is unable to perform all tasks conjointly anymore – while still capable to achieve each test separately (11, 57) (**Figure 2**).

Regarding time pressure, in addition to the fact that the lack of any rest may give insight into a possible decrease of the sustained attention throughout the procedure, constraining the patient to perform the multi-tasking in less than 5 seconds also enhances the sensitivity of the functional monitoring. Indeed, in fMRI studies, time restriction has been demonstrated to increase cognitive effort and to enhance activity across the entire multiple-demand network (46). Importantly, the clinical implications of this time parameter have been evidenced, since naming speed has been significantly correlated to the return to work in patients who underwent LGG resection (31). Furthermore, it is worth noting that multi-tasking does not extend the duration of surgery, because by definition several tasks are performed simultaneously and not serially. Therefore, this approach is adapted to practical constraints in the operating theater, since it does not increase the risk of patient tiredness over time (9, 56) and does not require to inflate the amount of DES (therefore with no higher risk of intraoperative seizures).

Concerning the postoperative outcomes of LGG patients who benefited from intrasurgical multi-tasking in clinical routine, beyond an increase of the overall survival thanks to an optimization of the extent of resection (1, 2, 7), and besides the fact that the risk of long-term severe permanent neurological deficit is currently less than 1% (3, 4, 58), recent series in which objective neuropsychological assessments have systematically been performed after surgical resection demonstrated that a preservation or even an improvement of cognitive scores was found in most patients and should be considered as a standard of care – in patients with preoperative symptoms as well as in patients with incidental discovery of LGG (4, 59). Interestingly, sparing the interplay between networks during a first surgery opened the door to subsequent operation(s) owing to mechanisms of functional reshaping which occurred in the meantime (2, 60, 61), and resulted in the maximization of resection while maintaining neurocognition and therefore quality of life (62). Above all, the use of multi-tasking with time constraint in awake patient is associated with a higher rate of employment resumption (31), between 94% to 97% in recent experiences (3, 4, 58).

## Conclusions and Perspectives

These data support that a more naturalistic multi-tasking protocol achieved in the operating room, which reflects the critical dynamic interactions across parallel circuits acting together and not in isolation – to allow the emergence of complex context-specific behaviors - is able to give insights to promote the return to an active life adapted to the environmental demands for LGG patients undergoing awake surgical resection.

The next step would be to keep on the sensitivity of tasks performed during surgery and to tailor their complexity to more integrated functions and behaviors which constitute the essence of human being, even though still neglected by neurosurgeons and neurooncologists, including in terms of personality (18) and creativity (63). To this end, in addition to the peri-operative non-invasive functional neuroimaging, DES mapping represents a unique opportunity to decipher not only function-specific networks but also the cross-talk between neural circuits, enabling to improve the elaboration/selection of more ecological tasks and their optimal administration (order, sequence, combination, timing) throughout the resection in agreement to patients’ expectations and according to their vision of life.

## Data Availability Statement

The original contributions presented in the study are included in the article/supplementary material. Further inquiries can be directed to the corresponding author.

## Author Contributions

HD and GH contributed to conception and design of the study. HD wrote the first draft of the manuscript. All authors wrote sections of the manuscript. All authors contributed to manuscript revision, read, and approved the submitted version.

## Conflict of Interest

The authors declare that the research was conducted in the absence of any commercial or financial relationships that could be construed as a potential conflict of interest.

## Publisher’s Note

All claims expressed in this article are solely those of the authors and do not necessarily represent those of their affiliated organizations, or those of the publisher, the editors and the reviewers. Any product that may be evaluated in this article, or claim that may be made by its manufacturer, is not guaranteed or endorsed by the publisher.
